# Protective roles of erythropoiesis-stimulating proteins in chronic heart failure with anemia

**DOI:** 10.3892/etm.2014.1845

**Published:** 2014-07-14

**Authors:** SHUQIN ZHOU, YUGANG ZHUANG, WEI ZHAO, BOJIE JIANG, HUI PAN, XIANGYU ZHANG, HU PENG, YANQING CHEN

**Affiliations:** Department of Emergency Critical Care Medicine, Shanghai Tenth People’s Hospital of Tongji University, Shanghai 200000, P.R. China

**Keywords:** anemia, chronic heart failure, erythropoiesis-stimulating proteins, hospitalization, mortality

## Abstract

Anemia is a common comorbidity in patients with chronic heart failure (CHF) and is frequently treated with erythropoiesis-stimulating proteins (ESPs). Previous studies, however, have been relatively short in duration and have not provided conclusive data on the safety or clinical efficacy of ESP treatment. The aim of this study was to explore the safety and therapeutic effects of ESPs in patients with anemia and CHF. A systematic literature search in EMBASE and MEDLINE from their inception to July 2013 was performed, and clinical studies that evaluated the effects of ESPs among patients with CHF were identified. Randomized clinical trials comparing the effects of ESP treatment with those of placebo treatment or usual care regimes in anemic patients with CHF were included. Nine randomized, controlled trials were identified, comprising 750 patients with CHF and anemia receiving ESP treatment for between three months and one year. ESP treatment had a significantly lower risk of CHF hospitalization [relative risk (RR), 0.47; 95% confidence interval (CI), 0.32–0.70; P=0.0002] and a moderate reduction in mortality risk (RR, 0.68; 95% CI, 0.38–1.19; P=0.18). Treatment with ESPs in patients with symptomatic CHF and anemia resulted in significant improvements in hemoglobin, hematocrit and brain natriuretic peptide levels, as well as exercise capacity, renal function, New York Heart Association class and left ventricular ejection fraction. In conclusion, this study found that treatment with ESPs exerts beneficial effects against CHF and is not associated with a higher mortality rate or adverse effects. These outcomes support the instigation of a trial evaluating the treatment of anemia with ESPs in patients with chronic CHF.

## Introduction

Anemia is a common comorbidity in patients with chronic heart failure (CHF) (1–4) and is associated with increased morbidity. The pathogenesis of anemia in CHF is complex and is associated with a number of factors, including renal dysfunction, plasma volume fluctuation, inflammation, hematinic deficiencies and drug treatment (5). Initial data from a large observational study of patients with CHF attending cardiology clinics showed that 33% had a low hemoglobin (Hb), using the most commonly employed definition of the World Health Organization (5). Notably, anemia has been described as an independent predictor of the risk of mortality and hospitalization due to diastolic dysfunction and left ventricular (LV) dysfunction in patients with CHF; diastolic and LV dysfunction may both result in disability or mortality (6–8).

A number of preliminary studies have reported that the correction of low Hb concentrations using erythropoiesis-stimulating proteins (ESPs) may improve cardiac and renal function and reduce the requirement for hospitalization and diuretics in patients with CHF (9–10). Erythropoietin (EPO) is an erythropoiesis-stimulating, 165-amino acid glycoprotein hormone secreted by the kidney in response to hypoxia and has a pivotal role in promoting an increase in red blood cells. EPO acts by stimulating the generation and release of cells from the bone marrow, thus improving the oxygen-carrying capacity of the blood. EPO performs additional functions beyond that of hematopoiesis, including cardioprotection (11).

Most interventional studies conducted in patients with CHF and anemia have shown an improvement in exercise tolerance and functional status in response to treatment; however, these were uncontrolled studies or controlled studies without a placebo. Furthermore, these studies were relatively short in duration and not designed to provide conclusive data on the safety or clinical efficacy of ESP treatment. In addition, the small sample sizes produced underpowered results. In this study, a meta-analysis was performed to explore the safety and therapeutic effects of ESPs in patients with anemia and CHF.

## Materials and methods

### Selection of published studies

The databases EMBASE (www.embase.com) and MEDLINE (www.ncbi.nlm.nih.gov/pubmed) were searched from their inception to July 2013, and clinical studies that evaluated the effects of ESPs among patients with CHF were systematically identified. No language restriction was applied. Search terms included ‘heart failure’ or ‘congestive heart failure’ or ‘chronic heart failure’ or ‘CHF’ combined with ‘recombinant erythropoietin’ or ‘darbepoetin’ or ‘erythropoietin’ or ‘erythropoiesis’. The titles and abstracts of studies identified in the automated search were scanned to exclude any irrelevant articles. The full texts of the remaining articles were read to determine whether they contained relevant information on the topic of interest. Cited references of retrieved and review articles were assessed to confirm that the assembled list of relevant publications was complete.

**Figure 1:**
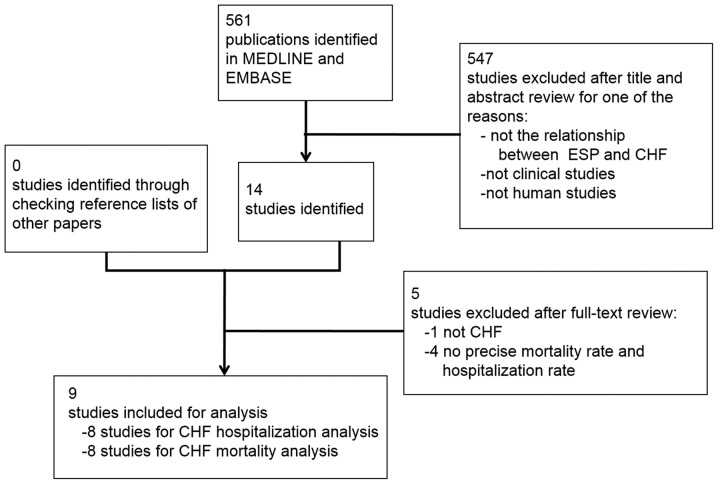
Study selection diagram. CHF, chronic heart failure; ESP, erythropoiesis stimulating protein.

Fig. 1 illustrates the search and selection process. The titles and abstracts of the primary 561 publications identified were reviewed and 547 were discarded for one of the following reasons: (i) The study did not discuss the association between ESPs and HF; (ii) the study was not a clinical study; (iii) the study was not a human study. Bibliographies were also searched for publications not identified in the database searches but no more additional publications were found. In total, the full texts of 14 articles were read. If the type of HF was not CHF (e.g. systolic HF) (12) or no precise mortality or hospitalization rate could be calculated (13–16), the publications were excluded. Nine publications were ultimately selected for the meta-analysis.

### Data extraction and quality assessment

The eligibility of all studies retrieved from the databases was independently evaluated and the relevant data from each study were extracted using a unified data form. The items included in the data form were as follows: (i) Study name (with first author’s name and year of publication); (ii) journal name; (iii) location; (iv) study design; (v) study population (case and control); (vi) inclusion criteria; (vii) exclusion criteria; (viii) range for follow-up; (ix) end-points. Two separate lists from two independent authors were compared, and disagreements were resolved by consensus. Relative risks (RRs) were recorded or calculated.

Each study was evaluated for quality according to the guidelines provided by the United States Preventive Task Force. The following characteristics were assessed: (i) Duration of follow-up (>6 months); (ii) adequacy of follow-up (reporting loss rate of follow-up); (iii) definition of anemia; (iv) full specification of outcome; (v) study sample representative for the unexposed population; (vi) full specification of clinical and demographic variables; (vii) explanation of sample selection; (viii) clear inclusion and exclusion criteria. Studies were graded as ‘poor’ quality if they met <5 criteria, ‘fair’ if they met 5–6 criteria and ‘good’ if they met >7 criteria. Outcomes assessed were mortality and CHF hospitalization rate.

### Statistical analysis

To compute a summary RR with a 95% confidence interval (CI), a study-specific, most-adjusted RR and its 95% CI was used in all analyses. Publication bias was assessed by visual inspection of the funnel plots created by plotting the RR to standard error (SE) for all nine studies.

Inter-study heterogeneity was examined using Cochran’s Q and I^2^ statistics. The I^2^ statistic assesses the percentage of variability in the effect estimates that is due to heterogeneity rather than chance. A fixed-effects model was used if P<0.10 or I^2^ >50% for heterogeneity, otherwise a random-effects model was utilized. The RR was transformed to a natural log scale and the SEs were calculated. The analysis was performed using RevMan 5.2 software (The Nordic Cochrane Centre, The Cochrane Collaboration, Copenhagen, 2012).

## Results

### Literature search, study characteristics and quality assessment

Following the exclusion of duplicates, 561 potentially relevant studies were reviewed, with nine clinical studies meeting the inclusion criteria and subsequently being analyzed (Fig. 1). The baseline characteristics of the studies included are summarized in Table I. The nine studies, published between 2001 and 2011, followed 750 patients with CHF and anemia receiving ESP treatment for between three months and one year. The inclusion criteria for anemia differed from Hb (17–23) to hematocrit (Hct) (24). Five studies used EPO as the ESP, while the remaining four used darbepoetin-α. All studies were conducted in Europe with a ‘fair’ quality score.

**Figure 2:**
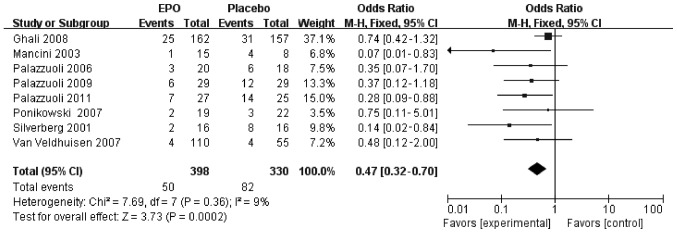
Pooled risk ratio for hospitalization in randomized, placebo-controlled trials of erythropoiesis-stimulating protein treatment in patients with heart failure and anemia. CI, confidence interval; M-H, Mantel Haenszel; EPO, erythropoietin.

Eight of the nine studies reported detailed CHF hospitalization. Of the 398 patients with CHF in the ESP treatment group, 50 were admitted to hospital (12.6%), whilst 82 of the 330 placebo-treated patients were hospitalized (24.8%). A significantly reduced hospitalization risk was identified for patients with CHF treated with ESPs compared with those treated with placebo (RR, 0.47; 95% CI, 0.32–0.70; P=0.0002) (Fig. 2). With regard to the outcomes, no statistically significant differences in heterogeneity were found between the included studies for hospitalization (I^2^=9%, P=0.36), and there was no obvious indication of publication bias.

**Figure 3:**
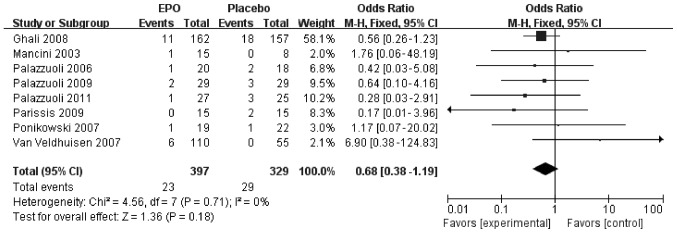
Pooled risk ratio for mortality in randomized, placebo-controlled trials of erythropoiesis-stimulating protein treatment in patients with heart failure and anemia. CI, confidence interval; M-H, Mantel Haenszel; EPO, erythropoietin.

Eight of the nine studies reported details of CHF mortality rate. Of the 397 patients with CHF in the ESP treatment group, 23 patients succumbed during the study (5.8%), compared with 29 of the 329 placebo-treated patients (8.8%). This resulted in a moderate reduction, without reaching statistical significance, in mortality risk for patients with CHF treated with ESPs (RR, 0.68; 95% CI, 0.38–1.19; P=0.18) compared with those treated with placebo (Fig. 3). No statistically significant differences in heterogeneity were found between the included studies for hospitalization (I^2^=0%, P=0.71), and there was no obvious indication of publication bias.

The baseline and achieved Hb and Hct levels and red blood cell count of the different studies are shown in Table II. Patients treated with ESPs showed enhanced recovery from anemia (P<0.05). Furthermore, patients with CHF treated with ESPs exhibited improved exercise capacity with regard to exercise duration, volume of oxygen consumption and distance walked during exercise, as compared with controls (Table III). Following treatment with ESPs, a reduction in brain natriuretic peptide (BNP) and creatinine was observed in patients with CHF compared with controls (Table IV). It was additionally detected that ESP treatment significantly improved the New York Heart Association (NYHA) class and slowed the deterioration of LV ejection fraction (LVEF) in patients with CHF (Table V).

## Discussion

EPO receptors are expressed in both hematopoietic and nonhematopoietic cells. ESPs, which are clinically used exclusively for erythropoiesis in patients with anemia, demonstrate potential in the treatment of pathological conditions of nonhematopoietic organs, including the brain and heart (3). Further investigations have demonstrated that ESP-mediated cardioprotection is mainly achieved through reducing apoptosis, increasing neovascularization, mobilizing endothelial progenitor cells and inducing angiogenesis through the phosphorylation and activation of signaling pathways (7), including Janus kinase 1/2, signal transducer and activator of transcription 3 (STAT3), STAT5 and phosphoinositide-3-kinase (25).

Patients with CHF together with anemia experience higher rates of hospitalization than those without anemia. A prior meta-analysis by van der Meer *et al* (26) of seven prospective, randomized, placebo-controlled trials of the use of ESPs in CHF enrolling 650 patients suggested a statistically significant decrease of 41% in hospitalizations due to HF in ESP-treated patients compared with controls. A similar result was noted in the present study, and was consistent with a pooled, patient-level analysis of three randomized trials of darbepoetin α that enrolled 516 subjects with HF (27).

In the present pooled analysis of patients with HF, it was found that the anemia of patients with CHF improved with ESP treatment. The majority of the changes in the anemic condition occurred together with increases in Hb and Hct; however, the most significant improvements were observed after one year. A moderate reduction in mortality risk was also observed for patients with CHF treated with ESPs, compared with those treated with placebo. This may be due in part to the improved oxygenation and reduced oxidative stress caused by the improvement in the anemia or due to the direct effects of the drug. Another meta-analysis including >150,000 patients with CHF identified that low Hb levels increased the risk of all-cause mortality over a six-month to five-year follow-up period, irrespective of whether CHF was due to systolic or diastolic dysfunction (28). Other research, however, has reported contradictory results (26). Differences may be due to skews in data caused by patients with a poor initial response to the ESP, who went on to receive higher doses of the drug and had increased rates of cardiovascular events and all-cause mortality. The present study found that patients who received ESP treatment exhibited a statistically significant improvement in exercise capacity and confirmed a previous report, according to which subcutaneous EPO administration improves exercise capacity, quality of life and LVEF in patients with CHF and anemia (29).

BNP levels are a reflection of ventricular stretching and are accepted as an effective marker of the presence and severity of CHF, since high levels of BNP are independent predictors of adverse clinical outcomes in CHF (22). Neurohormonal and metabolic effects induced by anemia can result in direct myocardial toxicity, myocardial hypertrophy, and salt and water retention. A reduction in BNP levels in patients with CHF treated with ESPs was observed in the present study. This decrease in BNP levels could be associated with improvements in a number of aspects, including improvements in cardiac function as a result of increased oxygen supply to the heart, reduced load caused by the prevention of anemia-induced tachycardia and increased stroke volume, reduction in plasma volume and a reduction in the activity of the sympathetic and renin angiotensin-aldosterone system, which occurs in anemia (3,30). In the present study, the improvement in renal function in the treated group was reflected by the improvement in creatinine level. Since renal failure is one of the primary negative prognostic factors for mortality and morbidity in CHF, the preservation of renal function by reversal of anemia is another important contribution of ESP treatment for improved prognosis in CHF (5). In this context, the data demonstrated a trend towards a reduction in cardiac adverse events and a significant improvement in NYHA class and LVEF without any other potential adverse effects.

In conclusion, treatment of patients with symptomatic CHF and anemia with ESPs results in significant improvements in hospitalization rate, Hb, Hct and BNP levels, mortality, exercise capacity, renal function, NYHA class and LVEF. These results support the instigation of larger trials investigating the safety and efficacy of ESPs for the treatment of anemia in anemic patients with symptomatic CHF.

## Figures and Tables

**Table I:** Summarized information of included studies.

First author (year)	Study design	No. of patients (ESP/Placebo)	Inclusion criteria	Exclusion criteria	ESP	Follow-up	Quality
Silverberg (2001)	Single-center study, randomized, open-label	32 (16/16)	LVEF: 40% Hb: 10–11.5 g/dl	Secondary causes of anemia	EPO	8.2 months	Fair
Mancini (2003)	Single-center study, randomized, single-blind, placebo-controlled	23 (15/8)	NYHA: III/IV Hct <35%	Non-ambulatory patients, continuous inotropic agents, iron deficiency anemia, sCr >2.5 mg/dl	α-EPO	3 months	Fair
Palazzuoli (2006)	Single-center study, randomized, double-blind, placebo-controlled	38 (20/18)	NYHA: III/IV Hb <11 g/dl	Secondary causes of anemia, isolated diastolic HF, <12 weeks MI, sCr <5.0 mg/dl	β-EPO	3 months	Fair
Ponikowski (2007)	Multi-center study, randomized, double-blind, placebo-controlled	41 (19/22)	Symptomatic CHF LVEF <40% Hb: 9–12 g/dl	Blood transfusion or ESP within 12 weeks, sCr >3.0 mg/dl	α-DPO	26 weeks	Fair
Van Veldhuisen (2007)	Multi-center study, randomized, double-blind, placebo-controlled	165 (56 WD+54 FD/55)	Symptomatic CHF LVEF <40% Hb: 9–12.5 g/dl	sCr >3.0 mg/dl	α-DPO	26 weeks	Fair
Ghali (2008)	Multi-center study, randomized, double-blind, placebo-controlled	319 (162/157)	NYHA: II–IV LVEF <40% Hb <12.5 g/dl	sCr >3.0 mg/dl	α-DPO	1 year	Fair
Parissis (2009)	Single-center study, randomized, single-blind, placebo-controlled	30 (15/15)		sCr >2.5 mg/dl	α-DPO	3 months	Fair
Palazzuoli (2009)	Single-center study, randomized, placebo-controlled, double-blind	58 (29/29)	NYHA: III–IV, Hb <11.5 g/dl, LVEF <40%	Intestinal bleeding, gastric lesions, vitamin B12 deficiency, severe renal failure	β-EPO	1 year	Fair
Palazzuoli (2011)	Single-center study, randomized, double-blind, placebo-controlled	42 (13 α-EPO+14 β-EPO/25)	NYHA: III–IV Hb <11.5 g/dl Cr clearance: 30–60 ml/min	Isolated diastolic dysfunction, more than moderate valvular disease, recent MI, modifiable causes of anemia severe renal failure, gastrointestinal bleeding	α-EPO/β-EPO	1 year	Fair

FD, fixed dose; WD, weight-adjusted dose; EPO, erythropoietin; ESP, erythropoiesis-stimulating protein; LVEF, left ventricular ejection fraction; NYHA, New York Heart Association; Hct, hematocrit; Hb, hemoglobin; CHF, chronic heart failure; Cr, creatinine; sCr, Serum Cr; MI, myocardial infarction; DPO, darbepoetin.

**Table II:** Comparison of anemia following ESP treatment.

First author (year)	No. of patients (ESP/Placebo)	Baseline Hb (g/dl)		Hb at end (g/dl)		Baseline Hct (g/dl)		Hct at end (%)		Baseline RBCs (1/ml)		RBCs at end (x10^x^/ml)	
					
		ESP	Placebo	ESP	Placebo	ESP	Placebo	ESP	Placebo	ESP	Placebo	ESP	Placebo
Silverberg (2001)	32 (16/16)	10.3	10.9	12.9	10.8								
Mancini (2003)	23 (15/8)	11.0	10.9	14.3	11.5								
Palazzuoli (2006)	38 (20/18)	10.4	10.6	12.4	10.5	30.0	32.0	36.4	31.0	3.3	3.4	4.2	3.2
Ponikowski (2007)	41 (19/22)	11.8	11.6	13.9	12.3								
Van Veldhuisen (2007)	165 (56 WD+54 FD/55)	11.5	11.4	13.3	11.4								
Ghali (2008)	319 (162/157)	11.5	11.3	13.6	11.8								
Parissis (2008)	32 (21/11)	11.0	11.4	12.8	11.8								
Kourea (2008)	41 (21/20)	10.9	11.4	12.8	11.7								
Palazzuoli (2009)	58 (29/29)	9.6	9.3	11.9	10.5	30.8	31.6	34.1	32.3	3.2	3.2	3.8	3.3
Parissis (2009)	30 (15/15)	11.2	11.5	12.8	11.9								
Palazzuoli (2011)	42 (13 α-EPO/14 β-EPO/25)	10.4/9.0	9.3	12.3/11.7	10.6	30.6/30.8	31.6	34.2/34.0	32.3	3.6/3.2	3.2	3.9/3.8	3.3

ESP, erythropoiesis-stimulating protein; WD, weight-adjusted dose; FD, fixed dose; RBC, red blood cells; Hb, hemoglobin; Hct, hematocrit; EPO, erythropoietin.

**Table III:** Comparison of exercise capacity following ESP treatment.

First author (year)	No. of patients (ESP/Placebo)	Baseline exercise duration (sec)		Exercise duration at end (sec)		Baseline VO_2_ (ml/kg/min)		VO_2_ at end (ml/kg/min)		Baseline distance walked (m)		Distance walked at end (m)	
					
		ESP	Placebo	ESP	Placebo	ESP	Placebo	ESP	Placebo	ESP	Placebo	ESP	Placebo
Mancini (2003)	23 (15/8)	590	542	657	459	11.0	10.0	12.7	9.5	362	283	405	321
Palazzuoli (2006)	38 (20/18)	348	348	468	360	12.8	12.5	15.1	12.0	278	285	356	266
Ponikowski (2007)	41 (19/22)	559	597	526	468	12.4	12.2	12.4	11.7				
Ghali (2008)	319 (162/157)	408	409	465.3	455.5								
Van Veldhuisen (2007)	165 (56 WD+54 FD/55)									287	304	321	315
Parissis (2008)	32 (21/11)									227	214	296	167
Kourea (2008)	41 (21/20)									201	237	274	204
Parissis (2009)	30 (15/15)									227	214	296	167

WD, weight-adjusted dose; FD, fixed dose; ESP, erythropoiesis-stimulating protein; VO_2_, oxygen volume.

**Table IV:** Comparison of BNP and creatinine following ESP treatment.

First author (year)	No. of patients (ESP/Placebo)	Baseline BNP (pg/ml)		BNP at end (pg/ml)		Baseline creatinine (mg/dl)		Creatinine at end (mg/dl)	
			
		ESP	Placebo	ESP	Placebo	ESP	Placebo	ESP	Placebo
Palazzuoli (2006)	38 (20/18)	568	585	271	496	2.5	2.4	1.8	2.2
Parissis (2008)	32 (21/11)	1102	788	661	1212				
Kourea (2008)	41 (21/20)	829	725	517	1040				
Parissis (2009)	30 (15/15)	1105	988	669	1202				
Palazzuoli (2009)	58 (29/29)	702	690	422	535	2.3	2.4	2.2	2.4
Palazzuoli (2011)	42 (13 α-EPO+14 β-EPO/25)	512/659	610	335/449	582	2.3/2.3	2.3	2.0/2.2	2.3
Silverberg (2001)	32(16/16)					1.7	1.4	1.7	1.8
Van Veldhuisen (2007)	165 (56 WD+54 FD/55)					1.4	1.5	1.3	1.6

BNP, brain natriuretic peptide; ESP, erythropoiesis-stimulating protein; EPO, erythropoietin; WD, weight-adjusted dose; FD, fixed dose.

**Table V:** Comparison of heart function following ESP treatment.

First author (year)	No. of patients (ESP/Placebo)	Baseline NYHA class		NYHA class at end		Baseline LVEF (%)		LVEF at end (%)	
			
		ESP	Placebo	ESP	Placebo	ESP	Placebo	ESP	Placebo
Palazzuoli (2006)	38 (20/18)	3.5	3.4	2.8	3.6				
Parissis (2008)	32 (21/11)	2.8	2.7	2.1	3.2	26	28	31	25
Palazzuoli (2009)	58 (29/29)	3.35	3.32	2.77	3.28	30.1	30.9	32.3	30.9
Palazzuoli (2011)	42 (13 α-EPO/14 β-EPO/25)	3.4/3.5	3.3	2.7/2.8	3.2				
Van Veldhuisen (2007)	165 (56 WD+54 FD/55)					29	27	28.98	28.27
Kourea (2008)	41 (21/20)					26	28	32	28
Parissis (2009)	30 (15/15)					28	27	33	28

WD, weight-adjusted dose; FD, fixed dose; ESP, erythropoiesis-stimulating protein; LVEF, left ventricular ejection fraction; NYHA, New York Heart Association; EPO, erythropoietin.
